# Supplementary material for: A Likelihood Approach for Real-Time Calibration of Stochastic Compartmental Epidemic Models
Source: PLoS Comput Biol. 2017 Jan 17;13(1):e1005257. doi: 10.1371/journal.pcbi.1005257 (PMC5240920; doi:10.1371/journal.pcbi.1005257)
Supplement: S1 File — (TAR.GZ) [file pcbi.1005257.s014.tar.gz › HSPH_Online-SI-Revision/PF08/n1-mild/n1-mild_PF2_table8.pdf]

1	newly 3 weeks spec	9.	7.62	{0., 18.}	67.5556	0.583175	newly 3 weeks cumul	15.	28.48	{5., 58.}	122.933	0.421028	newly 5 weeks spec	11.	6.09	{0., 15.}	68.0909	0.809316	newly 5 weeks cumul	33.	41.87	{5., 92.}	82.2727	0.433102	newly 47 weeks cumul	75.	83.1	{5., 224.}	89.28	0.561828
2	newly 3 weeks spec	1.	9.63	{0., 23.}	897.	0.883512	newly 3 weeks cumul	7.	29.86	{3., 58.}	346.	0.538527	newly 5 weeks spec	1.	7.47	{0., 18.}	687.	0.781043	newly 5 weeks cumul	9.	45.32	{4., 97.}	421.556	0.580791	newly 43 weeks cumul	9.	101.73	{5., 258.}	1044.78	0.79824
3	newly 3 weeks spec	7.	8.91	{0., 21.}	103.	0.607993	newly 3 weeks cumul	17.	31.64	{2., 71.}	138.588	0.52822	newly 5 weeks spec	1.	6.46	{0., 14.}	602.	0.843453	newly 5 weeks cumul	22.	45.58	{2., 106.}	159.636	0.570112	newly 52 weeks cumul	22.	87.74	{2., 221.}	342.727	0.701598
4	newly 3 weeks spec	14.	6.52	{0., 17.}	67.4286	0.861893	newly 3 weeks cumul	32.	23.29	{1., 56.}	65.5313	0.60986	newly 5 weeks spec	6.	4.7	{0., 14.}	81.	0.778852	newly 5 weeks cumul	48.	34.22	{1., 88.}	67.2083	0.656575	newly 46 weeks cumul	263.	67.01	{1., 193.}	75.6084	1.10848
5	newly 3 weeks spec	5.	4.77	{0., 11.}	76.6	0.605144	newly 3 weeks cumul	17.	16.72	{0., 37.}	70.7059	0.547476	newly 5 weeks spec	1.	3.59	{0., 10.}	327.	0.719436	newly 5 weeks cumul	20.	24.34	{0., 58.}	86.8	0.564642	newly 48 weeks cumul	23.	55.55	{0., 149.}	202.652	0.689724
6	newly 3 weeks spec	3.	22.49	{6., 42.}	650.333	0.766831	newly 3 weeks cumul	20.	72.4	{23., 129.}	265.3	0.489898	newly 5 weeks spec	7.	17.39	{3., 36.}	177.857	0.453217	newly 5 weeks cumul	35.	109.83	{31., 201.}	220.543	0.435872	newly 52 weeks cumul	116.	223.83	{38., 475.}	121.905	0.347744
7	newly 3 weeks spec	5.	8.12	{0., 21.}	127.2	0.63523	newly 3 weeks cumul	17.	27.77	{1., 63.}	119.588	0.556619	newly 5 weeks spec	6.	5.43	{0., 15.}	90.5	0.760044	newly 5 weeks cumul	25.	39.86	{1., 92.}	118.4	0.58725	newly 51 weeks cumul	45.	68.19	{1., 178.}	116.644	0.652355
8	newly 3 weeks spec	13.	5.52	{0., 14.}	66.	0.946438	newly 3 weeks cumul	40.	18.72	{0., 42.}	61.	0.807885	newly 5 weeks spec	15.	4.35	{0., 12.}	73.9333	1.13513	newly 5 weeks cumul	63.	27.84	{0., 66.}	62.6349	0.869483	newly 43 weeks cumul	262.	54.64	{0., 145.}	79.5802	1.30236
9	newly 3 weeks spec	0.1	4.45	{0., 11.}	4406.	1.18433	newly 3 weeks cumul	7.	14.71	{1., 32.}	161.857	0.556092	newly 5 weeks spec	0.1	3.45	{0., 11.}	3416.	1.02589	newly 5 weeks cumul	7.	21.61	{1., 53.}	250.714	0.601322	newly 42 weeks cumul	7.	41.73	{1., 101.}	531.	0.73349
10	newly 3 weeks spec	0.1	5.5	{0., 14.}	5442.	1.32057	newly 3 weeks cumul	1.	18.22	{1., 42.}	1736.	1.09053	newly 5 weeks spec	0.1	3.5	{0., 11.}	3476.	0.994052	newly 5 weeks cumul	1.	26.18	{1., 63.}	2532.	1.20314	newly 43 weeks cumul	1.	49.33	{1., 138.}	4847.	1.35607
11	newly 3 weeks spec	14.	5.02	{0., 13.}	68.4286	0.93634	newly 3 weeks cumul	36.	16.96	{0., 40.}	62.8889	0.809423	newly 5 weeks spec	13.	3.93	{0., 10.}	72.2308	1.07554	newly 5 weeks cumul	55.	25.25	{0., 58.}	62.4545	0.860061	newly 42 weeks cumul	283.	46.13	{0., 123.}	83.7703	1.38496
12	newly 3 weeks spec	13.	4.58	{0., 11.}	67.3846	0.901728	newly 3 weeks cumul	24.	16.33	{0., 34.}	60.875	0.652862	newly 5 weeks spec	5.	3.42	{0., 9.}	74.	0.774957	newly 5 weeks cumul	44.	23.76	{0., 56.}	60.6818	0.753921	newly 43 weeks cumul	158.	43.59	{0., 107.}	73.7025	1.08184
13	newly 3 weeks spec	2.	2.35	{0., 6.}	125.5	0.770039	newly 3 weeks cumul	5.	8.82	{0., 18.}	148.	0.697443	newly 5 weeks spec	0.1	1.71	{0., 6.}	1724.	0.619349	newly 5 weeks cumul	5.	12.49	{0., 29.}	216.6	0.746874	newly 44 weeks cumul	5.	21.16	{0., 52.}	389.2	0.845074
14	newly 3 weeks spec	0.1	5.21	{0., 12.}	5158.	1.23164	newly 3 weeks cumul	3.	18.82	{0., 43.}	562.	0.778224	newly 5 weeks spec	0.1	3.94	{0., 10.}	3910.	1.03201	newly 5 weeks cumul	3.	27.01	{0., 59.}	832.333	0.880767	newly 48 weeks cumul	3.	50.42	{0., 149.}	1612.67	1.04474
15	newly 3 weeks spec	14.	11.55	{1., 27.}	60.7857	0.466347	newly 3 weeks cumul	34.	40.54	{9., 88.}	67.8824	0.355923	newly 5 weeks spec	7.	8.89	{0., 24.}	96.1429	0.542644	newly 5 weeks cumul	49.	59.32	{12., 132.}	71.3469	0.374717	newly 48 weeks cumul	104.	114.96	{14., 286.}	83.25	0.488333
16	newly 3 weeks spec	6.	7.07	{0., 16.}	90.5	0.606066	newly 3 weeks cumul	34.	24.45	{0., 52.}	58.7941	0.629859	newly 5 weeks spec	9.	4.94	{0., 12.}	65.3333	0.747692	newly 5 weeks cumul	52.	35.44	{0., 76.}	60.	0.679931	newly 46 weeks cumul	164.	66.32	{0., 157.}	67.2683	0.93451
17	newly 3 weeks spec	12.	10.64	{0., 23.}	65.	0.543368	newly 3 weeks cumul	46.	39.05	{6., 79.}	53.6739	0.394007	newly 5 weeks spec	14.	8.58	{0., 19.}	59.4286	0.655708	newly 5 weeks cumul	71.	57.38	{8., 122.}	54.2254	0.428859	newly 49 weeks cumul	150.	101.85	{8., 242.}	59.7533	0.551718
18	newly 3 weeks spec	9.	4.61	{0., 13.}	65.6667	0.817112	newly 3 weeks cumul	36.	17.73	{0., 38.}	60.25	0.757738	newly 5 weeks spec	12.	4.32	{0., 11.}	68.6667	0.979369	newly 5 weeks cumul	56.	26.48	{0., 57.}	59.4643	0.794529	newly 42 weeks cumul	105.	53.89	{0., 142.}	68.1429	0.900212
19	newly 3 weeks spec	9.	5.3	{0., 13.}	63.1111	0.713456	newly 3 weeks cumul	31.	20.02	{1., 46.}	61.0968	0.578738	newly 5 weeks spec	2.	3.77	{0., 12.}	166.5	0.716079	newly 5 weeks cumul	38.	27.63	{1., 63.}	64.1316	0.58528	newly 49 weeks cumul	38.	53.98	{1., 116.}	112.632	0.597744
20	newly 3 weeks spec	2.	7.72	{0., 19.}	321.	0.625221	newly 3 weeks cumul	25.	27.02	{5., 56.}	71.44	0.411158	newly 5 weeks spec	0.1	6.23	{0., 16.}	6176.	1.33175	newly 5 weeks cumul	27.	40.38	{6., 88.}	102.667	0.44101	newly 46 weeks cumul	27.	79.81	{7., 186.}	236.926	0.556492
21	newly 3 weeks spec	2.	9.41	{0., 22.}	404.5	0.678161	newly 3 weeks cumul	9.	31.09	{4., 71.}	266.556	0.480506	newly 5 weeks spec	1.	7.55	{0., 19.}	691.	0.796698	newly 5 weeks cumul	10.	47.53	{6., 113.}	391.7	0.569445	newly 46 weeks cumul	10.	128.17	{6., 352.}	1195.1	0.830551
22	newly 3 weeks spec	11.	10.26	{1., 22.}	66.	0.480608	newly 3 weeks cumul	38.	34.39	{5., 73.}	60.3421	0.408514	newly 5 weeks spec	8.	8.15	{0., 19.}	85.875	0.668042	newly 5 weeks cumul	55.	51.08	{6., 107.}	65.1636	0.444257	newly 48 weeks cumul	274.	99.33	{6., 258.}	65.5876	0.781832
23	newly 3 weeks spec	4.	5.22	{0., 11.}	103.	0.571298	newly 3 weeks cumul	24.	18.85	{1., 43.}	63.0417	0.50668	newly 5 weeks spec	9.	4.15	{0., 11.}	68.5556	0.867553	newly 5 weeks cumul	45.	27.68	{2., 59.}	59.5111	0.578098	newly 43 weeks cumul	68.	49.76	{2., 118.}	71.2647	0.654933
24	newly 3 weeks spec	9.	7.63	{0., 18.}	67.4444	0.607975	newly 3 weeks cumul	31.	26.06	{1., 56.}	61.8065	0.512689	newly 5 weeks spec	8.	5.2	{0., 12.}	63.75	0.738055	newly 5 weeks cumul	48.	37.91	{1., 78.}	59.8958	0.538085	newly 45 weeks cumul	112.	72.81	{1., 163.}	62.1161	0.667921
25	newly 3 weeks spec	8.	3.68	{0., 10.}	73.	0.910451	newly 3 weeks cumul	29.	12.41	{0., 30.}	65.4138	0.821931	newly 5 weeks spec	12.	3.15	{0., 10.}	78.75	1.18478	newly 5 weeks cumul	48.	18.69	{0., 48.}	68.0208	0.907719	newly 42 weeks cumul	155.	39.95	{0., 108.}	78.6258	1.21581
26	newly 3 weeks spec	3.	6.99	{0., 17.}	187.	0.580487	newly 3 weeks cumul	7.	24.33	{2., 51.}	271.286	0.551998	newly 5 weeks spec	0.1	5.11	{0., 14.}	5070.	1.19908	newly 5 weeks cumul	11.	35.08	{2., 74.}	248.182	0.550645	newly 45 weeks cumul	11.	69.64	{5., 186.}	556.364	0.706343
27	newly 3 weeks spec	13.	4.79	{0., 13.}	70.2308	0.998017	newly 3 weeks cumul	30.	15.89	{0., 41.}	63.9	0.759851	newly 5 weeks spec	5.	3.49	{0., 10.}	80.6	0.825412	newly 5 weeks cumul	42.	23.25	{0., 59.}	65.3571	0.788425	newly 42 weeks cumul	51.	43.29	{0., 119.}	85.5882	0.814336
28	newly 3 weeks spec	5.	9.4	{0., 22.}	148.	0.635337	newly 3 weeks cumul	18.	33.67	{1., 75.}	139.278	0.602054	newly 5 weeks spec	9.	7.53	{0., 20.}	76.5556	0.753819	newly 5 weeks cumul	31.	50.02	{1., 111.}	120.	0.628592	newly 48 weeks cumul	190.	92.45	{1., 227.}	63.4474	0.870335
29	newly 3 weeks spec	0.1	6.9	{0., 14.}	6820.	1.54932	newly 3 weeks cumul	5.	24.09	{3., 52.}	399.	0.609593	newly 5 weeks spec	0.1	5.56	{0., 12.}	5504.	1.33921	newly 5 weeks cumul	5.	36.07	{3., 77.}	635.4	0.743207	newly 45 weeks cumul	5.	74.23	{3., 192.}	1398.6	0.948482
30	newly 3 weeks spec	8.	5.45	{0., 12.}	73.375	0.739495	newly 3 weeks cumul	27.	20.05	{0., 46.}	73.3704	0.732886	newly 5 weeks spec	5.	4.25	{0., 11.}	94.2	0.866294	newly 5 weeks cumul	40.	29.28	{0., 66.}	73.15	0.770068	newly 49 weeks cumul	44.	58.34	{0., 113.}	117.545	0.797531
31	newly 3 weeks spec	0.1	13.33	{2., 29.}	13 238.	1.86648	newly 3 weeks cumul	0.1	41.45	{9., 83.}	41 350.	2.45404	newly 5 weeks spec	0.1	10.15	{0., 26.}	10 074.	1.61741	newly 5 weeks cumul	0.1	63.19	{10., 129.}	63 090.	2.61041	newly 47 weeks cumul	0.1	133.91	{12., 362.}	133 810.	2.83363
32	newly 3 weeks spec	17.	6.35	{0., 18.}	67.2353	0.921801	newly 3 weeks cumul	43.	21.88	{0., 49.}	60.093	0.754201	newly 5 weeks spec	10.	4.96	{0., 14.}	72.2	0.921584	newly 5 weeks cumul	62.	32.4	{0., 82.}	61.3548	0.792368	newly 45 weeks cumul	224.	67.73	{0., 187.}	72.3438	1.12432
33	newly 3 weeks spec	3.	7.37	{0., 17.}	201.	0.652482	newly 3 weeks cumul	15.	26.24	{1., 54.}	124.933	0.519162	newly 5 weeks spec	1.	5.39	{0., 15.}	491.	0.767943	newly 5 weeks cumul	19.	37.97	{1., 79.}	148.158	0.556537	newly 46 weeks cumul	20.	68.87	{1., 161.}	290.65	0.685626
34	newly 3 weeks spec	9.	7.38	{0., 21.}	77.5556	0.64821	newly 3 weeks cumul	32.	24.15	{1., 50.}	63.1563	0.568037	newly 5 weeks spec	6.	5.44	{0., 12.}	83.6667	0.703733	newly 5 weeks cumul	42.	35.9	{1., 83.}	67.9524	0.566999	newly 45 weeks cumul	93.	68.16	{1., 153.}	73.4194	0.704419
35	newly 3 weeks spec	10.	8.61	{1., 20.}	63.3	0.477964	newly 3 weeks cumul	37.	30.02	{4., 55.}	53.8378	0.428237	newly 5 weeks spec	4.	6.15	{0., 15.}	114.75	0.540384	newly 5 weeks cumul	48.	43.18	{7., 85.}	57.7917	0.435921	newly 47 weeks cumul	53.	76.52	{8., 167.}	101.17	0.497812
36	newly 3 weeks spec	0.1	4.9	{0., 13.}	4844.	1.28112	newly 3 weeks cumul	0.1	18.76	{1., 46.}	18 674.	1.93226	newly 5 weeks spec	0.1	3.44	{0., 9.}	3412.	1.01263	newly 5 weeks cumul	0.1	26.14	{1., 63.}	26 054.	2.06836	newly 49 weeks cumul	0.1	46.08	{1., 113.}	45 994.	2.23692
37	newly 3 weeks spec	5.	7.66	{0., 20.}	114.	0.572982	newly 3 weeks cumul	27.	26.04	{2., 49.}	64.8889	0.462175	newly 5 weeks spec	7.	5.46	{0., 16.}	71.7143	0.671336	newly 5 weeks cumul	39.	37.71	{4., 82.}	65.9744	0.483112	newly 45 weeks cumul	50.	74.96	{4., 204.}	116.8	0.585342
38	newly 3 weeks spec	2.	5.83	{0., 14.}	248.5	0.707664	newly 3 weeks cumul	11.	22.21	{1., 57.}	155.364	0.586852	newly 5 weeks spec	0.1	4.11	{0., 11.}	4076.	1.10009	newly 5 weeks cumul	12.	30.61	{1., 78.}	202.083	0.629957	newly 49 weeks cumul	12.	56.92	{1., 134.}	417.	0.778992
39	newly 3 weeks spec	4.	4.23	{0., 10.}	111.25	0.799189	newly 3 weeks cumul	11.	15.55	{0., 35.}	114.455	0.632699	newly 5 weeks spec	4.	3.38	{0., 9.}	105.	0.910874	newly 5 weeks cumul	27.	22.89	{0., 51.}	84.9259	0.760004	newly 47 weeks cumul	33.	39.98	{0., 102.}	118.	0.817445
40	newly 3 weeks spec	0.1	6.54	{0., 15.}	6486.	1.37639	newly 3 weeks cumul	1.	22.67	{0., 54.}	2189.	1.17435	newly 5 weeks spec	0.1	4.61	{0., 11.}	4568.	1.18063	newly 5 weeks cumul	1.	32.19	{0., 74.}	3141.	1.30073	newly 50 weeks cumul	1.	56.85	{0., 134.}	5607.	1.4606
41	newly 3 weeks spec	3.	9.06	{1., 21.}	236.	0.526771	newly 3 weeks cumul	11.	31.16	{3., 69.}	214.	0.489347	newly 5 weeks spec	0.1	5.7	{0., 13.}	5648.	1.25806	newly 5 weeks cumul	11.	43.91	{3., 90.}	325.545	0.57716	newly 51 weeks cumul	11.	72.49	{3., 178.}	583.727	0.726577
42	newly 3 weeks spec	0.1	6.61	{0., 17.}	6550.	1.41131	newly 3 weeks cumul	0.1	23.36	{1., 54.}	23 272.	2.05357	newly 5 weeks spec	0.1	4.43	{0., 11.}	4388.	1.16586	newly 5 weeks cumul	0.1	32.9	{1., 75.}	32 812.	2.17983	newly 50 weeks cumul	0.1	59.2	{1., 137.}	59 112.	2.34797
43	newly 3 weeks spec	0.1	5.14	{0., 11.}	5086.	1.29426	newly 3 weeks cumul	5.	19.27	{2., 43.}	312.2	0.601068	newly 5 weeks spec	0.1	4.19	{0., 12.}	4162.	1.05871	newly 5 weeks cumul	5.	27.96	{2., 57.}	484.8	0.715291	newly 52 weeks cumul	5.	48.36	{2., 124.}	892.8	0.842982
44	newly 3 weeks spec	3.	5.52	{0., 13.}	142.	0.56475	newly 3 weeks cumul	3.	19.2	{2., 42.}	554.667	0.688514	newly 5 weeks spec	0.1	3.55	{0., 8.}	3510.	1.11755	newly 5 weeks cumul	3.	27.32	{2., 64.}	821.333	0.804825	newly 44 weeks cumul	3.	50.7	{3., 124.}	1599.33	0.964624
45	newly 3 weeks spec	7.	6.24	{0., 16.}	76.5714	0.627094	newly 3 weeks cumul	21.	20.57	{1., 47.}	74.9048	0.553347	newly 5 weeks spec	2.	4.57	{0., 12.}	198.5	0.679322	newly 5 weeks cumul	25.	30.49	{1., 67.}	91.8	0.577999	newly 42 weeks cumul	30.	60.25	{1., 151.}	166.233	0.669255
46	newly 3 weeks spec	13.	12.43	{1., 25.}	56.0769	0.422061	newly 3 weeks cumul	34.	42.1	{7., 74.}	65.0588	0.330597	newly 5 weeks spec	14.	9.58	{0., 20.}	56.4286	0.580688	newly 5 weeks cumul	70.	62.22	{9., 117.}	49.3714	0.351189	newly 49 weeks cumul	231.	121.13	{12., 274.}	57.9264	0.561086
47	newly 3 weeks spec	8.	10.08	{0., 23.}	88.25	0.5553	newly 3 weeks cumul	29.	34.96	{3., 69.}	74.6897	0.470903	newly 5 weeks spec	9.	7.33	{0., 16.}	63.6667	0.62555	newly 5 weeks cumul	47.	51.29	{3., 108.}	67.8511	0.490532	newly 47 weeks cumul	220.	105.36	{3., 230.}	57.8545	0.733462
48	newly 3 weeks spec	9.	5.59	{0., 14.}	72.1111	0.815519	newly 3 weeks cumul	23.	18.5	{0., 47.}	76.	0.689829	newly 5 weeks spec	13.	4.14	{0., 12.}	74.6154	1.08589	newly 5 weeks cumul	45.	27.05	{0., 68.}	68.0667	0.771666	newly 42 weeks cumul	52.	50.01	{0., 150.}	92.6346	0.780481
49	newly 3 weeks spec	14.	8.27	{0., 18.}	61.5	0.709168	newly 3 weeks cumul	26.	28.02	{1., 65.}	78.3846	0.552534	newly 5 weeks spec	13.	6.29	{0., 17.}	66.6923	0.855519	newly 5 weeks cumul	51.	41.66	{1., 92.}	65.6471	0.612291	newly 46 weeks cumul	150.	83.61	{1., 204.}	63.7667	0.814771
50	newly 3 weeks spec	9.	5.81	{0., 12.}	61.	0.625777	newly 3 weeks cumul	30.	20.51	{1., 44.}	56.1667	0.563195	newly 5 weeks spec	4.	4.48	{0., 11.}	94.5	0.644884	newly 5 weeks cumul	48.	29.88	{2., 63.}	55.2917	0.591826	newly 43 weeks cumul	70.	57.49	{2., 153.}	70.9	0.643585
